# Variability in subthalamic nucleus targeting for deep brain stimulation with 3 and 7 Tesla magnetic resonance imaging

**DOI:** 10.1016/j.nicl.2021.102829

**Published:** 2021-09-16

**Authors:** Bethany R. Isaacs, Margot Heijmans, Mark L. Kuijf, Pieter L. Kubben, Linda Ackermans, Yasin Temel, Max C. Keuken, Birte U. Forstmann

**Affiliations:** a Integrative Model-based Cognitive Neuroscience Research Unit, University of Amsterdam, Amsterdam, The Netherlands; b Translational Neuroscience, School for Mental Health and Neuroscience, Maastricht University, Maastricht, the Netherlands; c Department of Neurology, Maastricht University Medical Centre, Maastricht, The Netherlands; d Department of Neurosurgery, Maastricht University Medical Centre, Maastricht, The Netherlands

**Keywords:** Deep brain stimulation, Field strength, Magnetic resonance imaging, Subthalamic nucleus, Targeting

## Abstract

Deep brain stimulation (DBS) of the subthalamic nucleus (STN) is an effective surgical treatment for Parkinson’s disease (PD). Side-effects may, however, be induced when the DBS lead is placed suboptimally. Currently, lower field magnetic resonance imaging (MRI) at 1.5 or 3 Tesla (T) is used for targeting. Ultra-high-field MRI (7 T and above) can obtain superior anatomical information and might therefore be better suited for targeting. This study aims to test whether optimized 7 T imaging protocols result in less variable targeting of the STN for DBS compared to clinically utilized 3 T images. Three DBS-experienced neurosurgeons determined the optimal STN DBS target site on three repetitions of 3 T-T2, 7 T-T2*, 7 T-R2* and 7 T-QSM images for five PD patients. The distance in millimetres between the three repetitive coordinates was used as an index of targeting variability and was compared between field strength, MRI contrast and repetition with a Bayesian ANOVA. Further, the target coordinates were registered to MNI space, and anatomical coordinates were compared between field strength, MRI contrast and repetition using a Bayesian ANOVA. The results indicate that the neurosurgeons are stable in selecting the DBS target site across MRI field strength, MRI contrast and repetitions. The analysis of the coordinates in MNI space however revealed that the actual selected location of the electrode is seemingly more ventral when using the 3 T scan compared to the 7 T scans.

## Highlights

• Neurosurgeons are stable in STN targeting regardless of MRI field strength.
• Neurosurgeons are stable in STN targeting regardless of MRI contrast.
• The targeted STN electrode location is more ventral using 3 T versus 7 T scans.

## 1 Introduction

Since its introduction in the 1990 s, deep brain stimulation (DBS) of the subthalamic nucleus (STN) has proven to be an effective surgical treatment for advanced Parkinson’s disease (PD) (Benabid et al., 2009, Limousin et al., 1995). STN DBS for PD is especially efficacious in treating otherwise refractory tremor, motor fluctuations and dyskinesias (Deuschl et al., 2006, Limousin and Foltynie, 2019). However, in spite of these positive outcomes, STN DBS has the potential to induce a number of side-effects including behavioral changes, cognitive impairments and speech, balance or gait problems (Frank et al., 2007, Parsons et al., 2006, Temel et al., 2006, Zarzycki and Domitrz, 2020). These side-effects may be a product of suboptimal placement of the DBS lead (Gilmore et al., 2017, Kloc et al., 2017, Petry-Schmelzer et al., 2019). Here we focus on the first of many procedural steps that can contribute to such suboptimal placement; stereotactic planning of the electrode site (Giller & Jenkins, 2015).

Targeting the STN can either be done using a constant coordinate relative to a given anatomical landmark or by visualizing the STN per individual and determining the target per patient. These two approaches are respectively called indirect and direct targeting, where direct targeting typically results in a better patient outcome as individual anatomical variability is taken into account (Lahtinen et al., 2020). Common clinical practices for direct targeting of the STN for DBS is achieved using T2-weighted (-w) magnetic resonance imaging (MRI) (Bus et al., 2018, Verhagen et al., 2016). T2-w MRI is sensitive to iron content, and the STN is rich in iron, which causes it to appear hypointense compared to the surrounding grey matter structures (Deistung et al., 2013, de Hollander et al., 2014). Additionally or alternatively, some centers incorporate intraoperative microelectrode recordings (MER) for target verification, while others rely exclusively on indirect targeting approaches with MER and standardized coordinate systems (Habets et al., 2019). Notably, most centers perform the DBS surgery in awake settings with local anaesthesia, thereby enabling the clinician to assess stimulation related side-effects during test stimulation and adjustment of the final electrode targeting. STN surgeries are increasingly being performed under general anaesthesia and therefore the precision of the stereotactic planning with MRI is becoming even more important.

Direct targeting for DBS traditionally relies on lower field MRI (1.5 and 3 T MRI) which are prone to low contrast and signal to noise ratios (CNR and SNR, respectively), and result in images that lack sharp and clear borders of small deep brain structures (Forstmann et al., 2017, Isaacs et al., 2020a). Ultra-high-field MRI systems (7 T and above) can obtain submillimeter anatomical information with increased contrast (Inglese et al., 2018, Keuken et al., 2018). Whether the benefits of ultra-high-field MRI result in better targeting for DBS remains unclear (Bot et al., 2018, Bot et al., 2019, Duchin et al., 2018, Hartmann et al., 2019, Isaacs et al., 2020b, Springer et al., 2016).

In addition to higher field strengths, quantitative imaging methods may contribute to the visualisation of DBS targets as they convey microstructural properties of the area of interest. For example, while T2* contrasts visualize the STN as a hypointense structure, they can provide additional quantitative maps that provide information in relation to iron content and load (Chavhan et al., 2009, Elolf et al., 2007, Plantinga, 2016). Further, effective transverse relaxation rate, or R2*, maps (R2*=1/T2*) derived from T2* contrasts are even more sensitive to iron load and visualize the STN as a hyperintense structure (Ulla et al., 2013). T2* contrasts can be processed into Quantitative Susceptibility Maps (QSM) which are also sensitive to iron. However, contrary to T2* and R2* based modalities, QSM accounts for local susceptibility inhomogeneities by incorporating both magnitude and phase image information as well as incorporating methods to remove background fields such as a dipole convolution (Schäfer et al., 2012, Zhou et al., 2014). This has led some groups to suggest that QSM is the superior contrast for imaging subcortical structures that are high in iron content (Alkemade et al., 2017, Isaacs et al., 2020b).

Once the STN is visualized the question still remains where to place the electrode. While the exact optimal site of stimulation within the STN is still under debate (e.g., Hamel et al., 2017) and seems to vary per patient (Horn et al., 2017, Vanegas-Arroyave et al., 2016), DBS is thought to be most effective in treating PD when the lead is placed in the dorsolateral (sensorimotor) portion of the nucleus (Duchin et al., 2018, Hamel et al., 2017, Starr, 2002, Welter et al., 2014). When targeting towards the ventral (limbic) portion of the STN, cognitive and psychiatric side-effects are more likely to occur (Machado et al., 2006). Or in the words of lead-DBS core-developer Andreas Horn ‘Millimetres matter’ when it comes to DBS (Horn et al., 2019). The importance of precision is highlighted by the recent work of (Schrock et al., 2021) where within-patient repositioning of the lead location in the STN resulted in marked improvement of motor symptoms and reduction of associative and cognitive side-effects. The precision of electrode target selection is therefore considered to be one of the first of many important factors that determine DBS outcome but the reproducibility, to the best of our knowledge, has not been formally investigated.

This study aims to test whether optimized 7 T imaging protocols including T2*, R2* and QSM contrasts result in less variable targeting for STN DBS than clinically utilized 3 T T2 scans. Three neurosurgeons targeted, what they considered the optimal STN DBS site, on 3 repetitions of 3 T-T2, 7 T-T2*, 7 T-R2* and 7 T-QSM images for five PD patients (Benabid et al., 2009). A low degree of variability across repetitions would indicate that the MR image allows for a consensus view as to the optimal target location, whereas a high degree of variability would indicate that the image lacks the required visibility to reach a unanimous agreement. We do not focus on the performance of the individual neurosurgeons, but we specifically focus on the amount of variability in the targeted coordinates of the various MR image modalities. We hypothesize that the test–retest reliability of STN targeting will be higher for the optimized 7 T contrasts than for the clinically utilized 3 T images. Further, we hypothesize that the test–retest reliability of STN targeting on 7 T-QSM contrasts will be higher compared to 7 T-T2* or R2* as previous literature has suggested that QSM is superior in imaging the STN at 7 T. A second aim is to test whether different MRI contrasts can result in different target locations as each MRI contrast contains complimentary anatomical information (Bazin et al., 2020, Visser et al., 2016a, Visser et al., 2016b).

## 2 Materials and methods

### 2.1 Participants

A total of five PD patients participated in the study (M = 4; F = 1) with a mean age of 62.2 years (SD = 7.9 years) and a mean number of 8.4 years since the official diagnoses (SD = 3.6 years). The number of patients in this study was limited due to the availability of patients meeting all the inclusion criteria and on the feasibility for the neurosurgeons to perform the stereotactic planning. PD patients were recruited as candidates for DBS surgery at the Neurology department within the Maastricht University Medical Centre (The Netherlands). The study was approved by the local Medical Ethical Committee at the Maastricht University Medical Centre (NL60342.068.17/METC172010). All data was collected and is held in accordance with the EU General Data Protection Regulation (GDPR) and the Dutch Act on Implementation of the GDPR, good clinical practice and relevant data protection laws. PD patients had no diagnosed neurological comorbidities and provided written informed consent prior to the scanning.

### 2.2 Data acquisition

#### 2.2.1 MRI acquisition

##### 2.2.1.1 3 Tesla MRI

Each PD patient underwent a preoperative clinical 3 T scan as part of the standard clinical practice with a Phillips Ingenia scanner using a 32-channel head coil at the Maastricht University Medical Center. The 3 T data that was obtained consisted of the standard clinical sequences used for DBS planning at the Maastricht University Medical Center. A whole brain 3D turbo field echo (TFE) T1-w scan was obtained with 1 mm isotropic voxel sizes, with the following parameters: Repetition Time (TR) = 8.1 ms, Echo Time (TE) = 3.7 ms, Inversion Recovery (IR) delay = 776 ms, Flip Angle (FA) = 8°, Bandwidths (BW) = 191.5 Hz/px, Echo Spacing (ES) = 13.6 ms, TFE factor = 183, transverse orientation acquisition in the anterior-posterior direction, with SENSE factor of 1.4 and total acquisition time (TA) of 05:51mins. A whole brain T2-w scan was obtained with spin echo sequence with 0.45 × 0.45 × 2 mm voxel sizes, with the following parameters: 65 slices, TR = 8264 ms, TE 80 ms, FA = 90°, BW = 193.6 Hz/px, TFE factor = 15, transverse orientation acquisition in the anterior-posterior direction, with SENSE factor of 1.5 and TA of 06:20mins.

##### 2.2.1.2 7 Tesla MRI

In addition to the standard clinical 3 T acquisition, a 7 T scan was acquired with a Siemens Magnetom scanner using a 32-channel head coil at the Scannexus Centre for Neuroimaging in Maastricht. Whole brain T1-w 3D images were obtained with an adapted version of the multi echo MP2RAGE (magnetization-prepared rapid gradient echo multi-echo) sequence (Caan et al., 2018, Marques et al., 2010) with 0.8 mm isotropic voxel sizes and the following parameters: 208 slices, TR = 6000 ms, TE _1,2_ = [2.74 ms, 8.71 ms], Inversion Time (TI) _1,2_ = [750 ms, 29000 ms], FA _1,2_ = [4°, 6°], BW _1,2_ = [350 Hz/Px, 150 Hz/Px], ES = 13.6 ms, interleaved and single shot multi slice mode and interleaved, sagittal orientation acquisition in the anterior-posterior direction, phase partial Fourier 6/8, parallel acquisition with GRAPPA and acceleration factor of 3 and TA of 10:56mins. Where possible, dielectric pads were placed between the side of the participants head and the receiver coil to reduce B_1_ inhomogeneity artefacts. The T2*-w 3D scan was acquired with a partial volume GRE (gradient echo) ASPIRE (multi-channel phase data from multi-echo acquisitions) sequence covering the subcortex with 0.5 mm isotropic voxel sizes and the following parameters: 90 slices, 16.7% slice oversampling, TR = 33 ms, TE _1-4_ = [2.49 ms, 6.75 ms, 13.50 ms, 20.75 ms], FA = 12°, BW _1-4_ = [300 Hz/px, 300 Hz/px, 200 Hz/px, 100 Hz/px], interleaved multi slice mode, sagittal orientation acquisition in the anterior-posterior direction, slice partial Fourier 7/8, parallel acquisition with GRAPPA and acceleration factor of 2 and TA 07.42mins (Eckstein et al., 2018).

#### 2.2.2 Calculation of quantitative MRI maps

All quantitative maps were created in native space. First, skull information was removed using the Brain Extraction Tool as implemented in FSL 5.0 (Jenkinson et al., 2012, Smith, 2002). The 3 T T2-w MRI sequence did not allow the calculation of quantitative maps due to the acquisition parameters. The maps for 7 T MRI scans were created using the following procedure: T2*-maps were computed by least-squares fitting of the exponential signal decay over the four echoes of magnitude image from the GRE ASPIRE sequence (Whittall et al., 1997). R2* maps were then calculated by taking the reciprocal of the T2* map. For QSM, phase maps of the fourth echo were pre-processed using iHARPERELLA (integrated phase unwrapping and background phase removal using the Laplacian) and used to calculate QSM with LSQR (sparse linear equation and least-squares method) (Li et al., 2014, Li et al., 2015, van Bergen et al., 2016).

#### 2.2.3 Targeting the STN

**Fig. 1:**
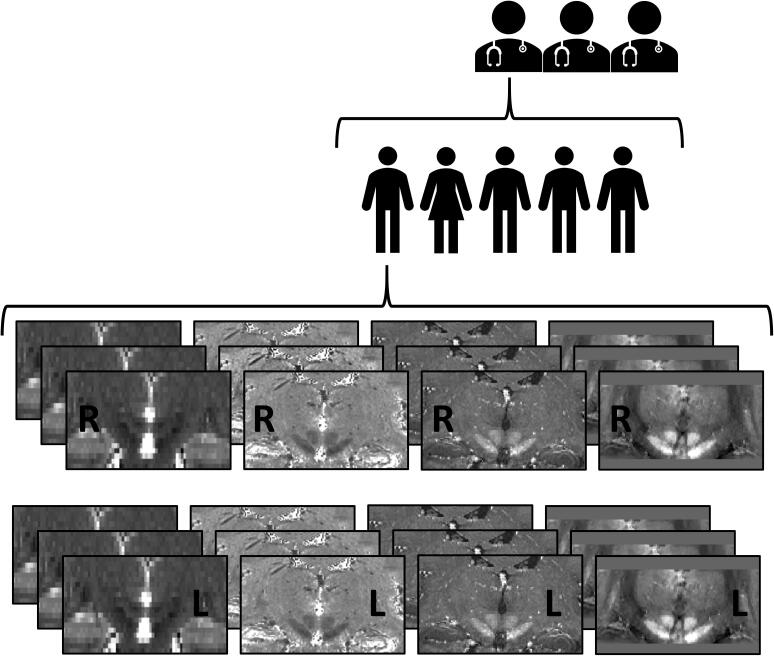
Targeting procedure of the subthalamic nucleus (STN). Identification was conducted by three neurosurgeons. Each neurosurgeon targeted the STN for five deep brain stimulation (DBS) candidates suffering from Parkinson’s Disease (PD), on four image modalities, with three repetitions, for both left and right STN. The image modalities included (from left to right) a 3 T-T2 weighted MRI, 7 T-T2* map, 7 T-R2* map and 7 T Quantitative Susceptibility Map (QSM). This resulted in a total of 120 STN targets per neurosurgeon, with 24 targets per patient.

Identification of the STN was conducted by a total of three neurosurgeons with a mean experience of 13.7 years (SD = 5.7 years) in STN DBS planning and surgery. Each neurosurgeon targeted separate left and right STNs per participant on the following image modalities: 3 T-T2, 7 T-T2*, 7 T-R2* and 7 T-QSM. All scans used to target the STN were in native acquisition space. The targeting procedure was repeated three times for every image and was assigned a novel identifier, so the neurosurgeons were unaware of the identification of each patient and repetition. The targeting procedure of the STN is shown in Fig. 1. Order of presentation of the images was fixed and the same for all three neurosurgeons. There were no images of the same participant following each other. Images were automatically loaded and presented in FSLeyes with pre-set intensity levels using an in-house Bash script. The masks were marked with the anonymized patient identifier, hemisphere and initial of the targeting neurosurgeon. The neurosurgeons then identified the coordinate in which they would place the DBS electrode, and a screenshot of this coordinate was saved. A total of 120 STN targets were obtained per neurosurgeon, and targeting was achieved in multiple sessions depending on the availability of the neurosurgeons. The first neurosurgeon was able to complete all targets in three sessions, with respectively 49 and 6 days between sessions. The second neurosurgeon completed the targeting in four different sessions that were spaced 67, 36, and 36 days apart. Finally, the third neurosurgeon finalized all STN targets in two sessions 13 days apart. This resulted in an average interval of 35 days between rating sessions, with a minimum of 6 days and a maximum of 67 days.

**Fig. 2:**
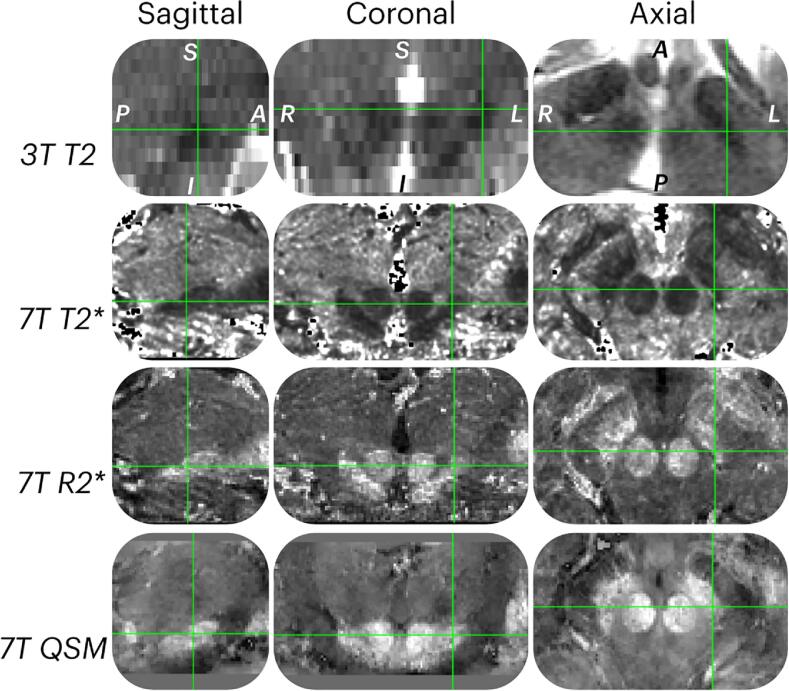
MRI image modalities. An example of each MRI image modality used for targeting for a single patient, including from top to bottom, a 3 T-T2 weighted MRI, 7 T-T2* map, 7 T-R2* map and 7 T Quantitative Susceptibility Map (QSM). The image shows a zoomed section of the subthalamic nucleus (STN) in the sagittal, coronal and axial planes. The STN is highlighted by the green intersection. (For interpretation of the references to colour in this figure legend, the reader is referred to the web version of this article.)

As the neurosurgeons were more used to planning on 3 T, instructions and examples were provided to explain the 7 T images with the following: i. ‘T2* images provide an indirect measure of iron content. Iron rich regions like the STN show a higher magnetic field perturbation compared to adjacent regions with lower iron content. The STN appears as a hypointense structure’. ii. ‘R2* maps offer a direct measure of magnetism. The STN appears as a hyperintense structure’. iii. ‘QSM (quantitative susceptibility maps) are post processed images based on the fourth echo of the T2* sequence, and invert the image contrast, also allowing for a direct measure of magnetism per voxel. The STN appears as a hyperintense structure’. The neurosurgeons were asked to define the position where they would place the electrode tip without taking the corresponding trajectory into account. An example of the intended electrode tip location for a patient by a single neurosurgeon is given in Fig. 2.

#### 2.2.4 Euclidean distance

The Euclidean distance (from here onwards called distance) between the repetitive coordinates was used as an index of variability, where smaller distances indicate better test–retest reliability (Liberti et al., 2014). The distance was calculated between the first and second repetition, second and third repetition, and first and third repetition. This resulted in three distance pairs per hemisphere for each contrast, subject, and neurosurgeon, or 90 distance pairs in total per MRI contrast. Due to technical errors six target coordinates were not saved correctly and are therefore missing from the dataset (resp. two 3 T coordinates and four 7 T coordinates), resulting in 348 distance pairs in total. To account for differences in voxel geometry between the 3 T and 7 T contrasts, the voxel coordinate of the target was transformed to millimetres by multiplying the x and y voxel coordinate values with, respectively, 0.44921875 or 0.53125 for the 3 T and 7 T coordinates and the z voxel coordinate values with, respectively, 2.0 or 0.5300006. This ensured that a direct comparison between the 3 T and 7 T derived distances was possible.

#### 2.2.5 Target coordinates in standard stereotactic MNI-space

To be able to compare the location of the target coordinate across subjects and MRI contrasts it was necessary to estimate the 3 T and 7 T slab transformations to standard MNI space. All individual scans were skull stripped using BET as implemented in FSL 5.0. The 3 T T2-w and the average of the four 7 T T2* volumes were registered to the 7 T whole brain T1-w scan using a rigid transformation ‘DenseRigid’ as implemented in ANTsPy. As the different sequences and field strengths have different levels of geometric distortions (Dammann et al., 2011, Duchin et al., 2012, Lau et al., 2018, Peerlings et al., 2019) the within-registration was also done using the non-linear symmetric normalization registration method ‘SyN’ as implemented in ANTsPy. This extra registration step was done to ensure that the results in MNI space were not driven by within-subject misalignment. The 7 T whole brain T1-w scan was registered to the icbm_avg_152_t1_tal_nlin_symmetric_VI 1 mm isotropic MNI template using the Symmetric normalization as implement in ANTsPy. This is a combination of affine and deformable transformations using mutual information as the optimization metric. All registration steps were visually inspected using the following landmarks: lateral ventricles, striatum, top indentation of the pons, corpus callosum and global outline of the brains. The landmarks were chosen for clear visibility between the different sequences and the location relative to the STN. Based on the alignment of the different landmarks, all registrations were considered to be reasonable. Note that all resulting registrations are visualized in the annotated Jupyter notebook. Using fslmaths a NifTi file was created for every single target coordinate in native space. The different transformation matrices were then combined with the deformation field and applied to the respective target coordinates using a bSpline interpolation. Finally, the X, Y and Z MNI coordinates of the Center of Gravity (COG) were extracted for every single target coordinate and used for further analyses. In line with our previous work (Keuken et al., 2017) we reduced the number of statistical tests by computing a principal component analysis (PCA) on the resulting X, Y, and Z COG coordinates. As we had no a-priori hypothesis regarding effects of lateralization and targeting precision, the negative X coordinates (corresponding to the left hemisphere) were converted to positive values before the PCA was calculated. The resulting first principal component corresponds to a new latent variable which captures the maximal amount of variance in the X, Y, and Z coordinates across the different target locations.

#### 2.2.6 Manual parcellation of the STN

The STN was manually parcellated by two independent anatomical experts (BRI and MCK) and verified by a third independent rater (MH), per patient, for both 3 T and 7 T images. The left and right hemispheres were parcellated separately. The 7 T parcellations were achieved by overlaying the 7 T-T2*, 7 T-R2* and 7 T-QSM contrasts together, to create a single 7 T parcellation based on the three image modalities. Parcellations were achieved in native space and were created to assess whether any differences in test–retest or MNI location could be explained by differences in STN visibility. This was quantified by calculating the Dice coefficient:

$$\mathrm{Dicecoefficient}=\frac{2*\left|m_{1}\cap m_{2}\right|}{\left|m_{1}\right|+\left|m_{2}\right|}$$

Where |m_i_| is the volume of the mask for rater _i_ and ${|m}_{1}\cap m_{2}|$ is the volume of the conjunct mask for rater 1 and 2. The conjunct mask therefore only includes the voxels in the STN that were included by both raters (Dice, 1945).

### 2.3 Data analysis

#### 2.3.1 Statistical methods

All statistical analyses were conducted using ANOVAs within a Bayesian framework using the JASP software package (V.0.14.1; (JASP Team, 2020)). The ANOVAs used a uniform prior model probability, and the assumption of normality were visualized using a Q-Q plot of the residuals. For both the test–retest reliability and the spatial location analyses patient ID and neurosurgeon ID were included as nuisance variables. For the Dice coefficient and volumetric analysis, the patient ID was included as a nuisance variable. The implementation of the Bayesian ANOVA in JASP relies on the R package BayesFactor (V.0.9.10–2; (Morey and Rouder, 2015, Rouder et al., 2012)). The resulting Bayes Factors (BF) are interpreted in light of assumptions proposed by (Jeffreys, 1998) and adapted by (Wetzels et al., 2011). Note that the analyses regarding the test–retest reliability, Dice coefficient and volume are based on values calculated in native space whereas the spatial location analysis is based on values in MNI space.

#### 2.3.2 Outlier analysis

Outliers were identified with the 1.5xIQR rule whereby any data point 1.5*IQR above the third quartile or below the first quartile was rejected from further analysis and was done per MRI contrast or field strength. For the distance pairs, 14 data points were identified as outliers across the MRI contrasts. The final sample for the test–retest ANOVA was 84 pairs for the 3 T-T2 contrast, 77 pairs for the 7 T-T2* contrast, 86 pairs for the 7 T-R2* contrast and 87 pairs for the 7 T-QSM contrast. For the coordinates in MNI space, there were two 7 T-T2* coordinates that were identified as outliers. There was a single 3 T Dice coefficient value and a single 7 T conjunction volume that were identified as outliers.

### 2.4 Open science

All target coordinates and STN parcellation masks are made available (DOI https://doi.org/10.17605/OSF.IO/DW2FR). In addition, an annotated Python notebook that was used to pre-process all the data and all resulting JASP files used to conduct the statistical analysis are provided.

## 3 Results

### 3.1 Test-retest reliability of the target coordinates.

**Fig. 3:**
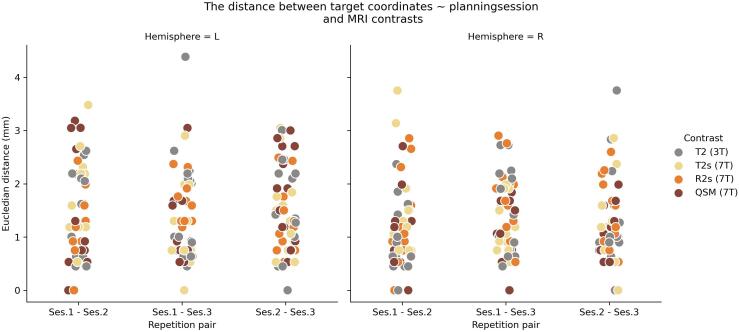
The Euclidean distance between the target coordinates over sessions and MRI contrasts. Note that we visualize the coordinates per hemisphere but as we had no a-priori hypothesis on lateralization, hemisphere was not included as a factor in the statistical testing.

On average the neurosurgeons deviated 1.35 mm (SD = 0.78) between sessions. In Table 1 the mean distances between the three targeting sessions are provided per MRI field strength and contrast whereas in Fig. 3 the distance between the pairs are visualized per hemisphere and MRI contrast.

**Table 1:** Targeting distance between the repetition pairs over MRI field strengths and contrasts.

MRI field strength	MRI Contrast	Repetition pairs	Mean (mm)	SD (mm)	N	Lower	Upper
3 T	T2	1–2	1.197	0.780	28	0.895	1.499
		1–3	1.554	0.934	29	1.198	1.909
		2–3	1.433	0.940	27	1.061	1.804
7 T	T2*	1–2	1.560	0.902	26	1.196	1.924
		1–3	1.178	0.661	24	0.899	1.457
		2–3	1.339	0.814	27	1.017	1.661
	R2*	1–2	1.170	0.715	28	0.893	1.447
		1–3	1.608	0.605	28	1.373	1.842
		2–3	1.370	0.623	30	1.138	1.603
	QSM	1–2	1.266	0.987	30	0.898	1.635
		1–3	1.061	0.623	28	0.819	1.302
		2–3	1.467	0.734	29	1.188	1.746

Note. The mean distance between sessions in millimetres and calculated over surgeon, patient and hemisphere.

#### 3.1.1 Model comparison.

To test whether MRI field strength, MRI contrast or planning session had an influence on the distance between target locations a Bayesian ANOVA was conducted. The primary output from the ANOVA is presented in Table 2, which shows the amount of support that the data offer for each model under consideration. The left-most column lists all models at hand: 18 alternative models and a single null model. The models are ordered by their predictive performance relative to the best model; this is indicated in the BF_10_ column, which shows the Bayes factor relative to the best model which, in this case is the Null model. For example, the data are 5.88 times more likely under the Null model than under the second-best model where MRI field strength (Tesla) is included as a predictor. This means that there is substantial evidence that there is no effect of field strength, MRI contrast or planning session on the test–retest reliability of the STN targeting.

**Table 2:** Model Comparison test–retest reliability of the target coordinates.

Models		P(M)		P(M|data)		BF _M_		BF _10_		error %
Null model (incl. PatientNr, Surgeon)		0.053		0.781		64.011		1.000		
T		0.053		0.133		2.751		0.170		4.842
RP		0.053		0.046		0.876		0.059		3.877
C		0.053		0.019		0.355		0.025		3.393
T + RP		0.053		0.007		0.133		0.009		1.875
T + C		0.053		0.007		0.124		0.009		2.249
T + C + T*C		0.053		0.003		0.046		0.003		7.390
T + RP + T*RP		0.053		0.002		0.029		0.002		4.812
C + RP		0.053		0.001		0.019		0.001		2.113
C + RP + C*RP		0.053		6.776e -4		0.012		8.681e -4		1.771
T + C + RP		0.053		3.902e -4		0.007		5.000e -4		2.594
T + C + RP + C*RP		0.053		2.620e -4		0.005		3.357e -4		3.145
T + C + RP + T*C		0.053		1.329e -4		0.002		1.703e -4		2.846
T + C + RP + T*C + C*RP		0.053		9.083e -5		0.002		1.164e -4		2.988
T + C + RP + T*RP		0.053		7.860e -5		0.001		1.007e -4		3.188
T + C + RP + T*RP + C*RP		0.053		5.930e -5		0.001		7.597e -5		2.295
T + C + RP + T*C + T*RP		0.053		2.653e -5		4.775e -4		3.398e -5		2.433
T + C + RP + T*C + T*RP + C*RP		0.053		2.356e -5		4.241e -4		3.019e -5		6.678
T + C + RP + T*C + T*RP + C*RP + R*C*RP		0.053		6.359e -6		1.145e -4		8.147e -6		2.739

Note.  All models include PatientNr, Surgeon.T: MRI field strength (Tesla); C: MRI Contrast; RP: Repetition pair; P(M): Prior model probability; P(M|data): posterior model probability; BF_M_: the change from prior odds to posterior odds; BF_10_: the Bayes factor relative to the best model; error %: indicates the precision of the numerical approximation and it is thought that in many situations an error percentage below 20.0% is acceptable (van den Bergh et al., 2020).

### 3.2 Spatial location of targets in MNI space.

**Fig. 4:**
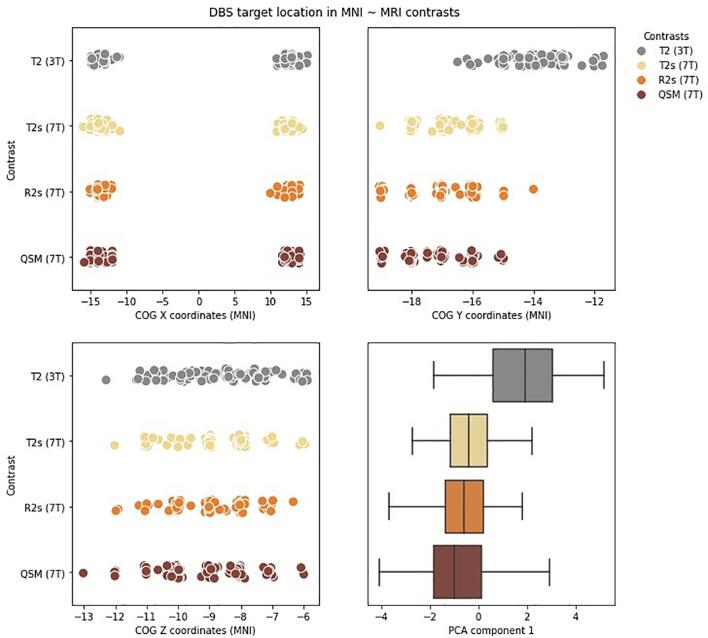
The intended DBS electrode location in MNI space over MRI contrasts. The left upper panel shows the X MNI coordinates of the planned electrode’s Centre of Gravity (COG) per MRI contrast, patient, surgeon and planning session registered from native to MNI space. Note that we visualize the X coordinates per hemisphere but as we had no a-priori hypothesis on lateralization, hemisphere was not included as a factor in the statistical testing. The right upper panel shows the Y MNI coordinates. The left lower panel shows the Z MNI coordinates. The right lower panel shows the boxplot of first PCA component per MRI contrast which were used for the statistical testing.

While we can conclude that the neurosurgeons are stable in selecting the electrode target over planning sessions it is unknown whether the neurosurgeons select similar targets across MRI field strengths and MRI contrasts. For that the individual electrode target locations were registered to MNI space and visualized in Fig. 4.

#### 3.2.1 Model comparison.

To test whether MRI field strength, MRI contrast or session had an influence on the DBS electrode location in MNI space a Bayesian ANOVA was conducted. The primary output from the ANOVA is presented in Table 3, which shows the amount of support that the data offer for each model under consideration. There is anecdotal evidence that the data is 1.74 times more likely under the model where MRI field strength is included than under the second-best model where MRI field strength and MRI contrast are included. There is however conclusive evidence that the data is more likely under the model including MRI field strength than under the Null model.

**Table 3:** Model comparison of the spatial location of the DBS electrode targets.

Models	P(M)	P(M|data)	BF _M_	BF _10_	error %
T	0.053	0.478	16.501	1.000	
T + C	0.053	0.274	6.804	0.574	5.736
T + C + T*C	0.053	0.130	2.682	0.271	4.329
C	0.053	0.044	0.823	0.091	2.914
T + R	0.053	0.035	0.645	0.072	3.366
T + C + R	0.053	0.022	0.400	0.045	8.444
T + C + R + T*C	0.053	0.009	0.170	0.020	4.263
C + R	0.053	0.003	0.059	0.007	3.861
T + R + T*R	0.053	0.002	0.045	0.005	3.162
T + C + R + T*R	0.053	0.001	0.026	0.003	4.968
T + C + R + T*C + T*R	0.053	7.006e −4	0.013	0.001	6.743
T + C + R + C*R	0.053	1.817e −4	0.003	3.798e −4	3.657
T + C + R + T*C + C*R	0.053	1.009e −4	0.002	2.111e −4	7.923
T + C + R + T*R + C*R	0.053	3.938e −5	7.089e −4	8.234e −5	21.787
C + R + C*R	0.053	3.166e −5	5.700e −4	6.620e −5	3.088
T + C + R + T*C + T*R + C*R	0.053	1.859e −5	3.346e −4	3.886e −5	21.086
T + C + R + T*C + T*R + C*R + T*C*R	0.053	2.288e −6	4.118e −5	4.784e −6	4.272
Null model (incl. PatientNr, Surgeon)	0.053	1.506e -41	2.711e -40	3.149e -41	2.182
R	0.053	6.767e -43	1.218e -41	1.415e -42	2.807

Note. All models include PatientNr, Surgeon.T: MRI field strength (Tesla); C: MRI Contrast; R: Repetition; P(M): Prior model probability; P(M|data): posterior model probability; BFM: the change from prior odds to posterior odds; BF10: the Bayes factor relative to the best model; error %: indicates the precision of the numerical approximation and it is thought that in many situations an error percentage below 20.0% is acceptable (van den Bergh et al., 2020).

As the amount of evidence to prefer the winning model over the second-best model was anecdotal, an analysis of effects was conducted (the results are given in Table 4). The BF_incl_ indicates that the data is 7.24 times more likely under the models that include MRI field strength than models without this predictor. Whereas the BF_incl_ indicates that the data is 2.98 times more likely under models that do not include MRI contrast as a predictor (1/0.336). This means that the target of the DBS electrode as quantified by the first component of the PCA differs between 3 T and 7 T MRI scans, where based on Fig. 4, this difference seems to be mainly along the Y-axis or in dorsal–ventral (brainstem orientation) | anterior-posterior (cerebrum orientation) direction. In the remainder we will use the brainstem orientation when referring to the MNI coordinate system.

**Table 4:** Analysis of Effects – spatial location of the DBS electrode targets.

Effects	P(incl)	P(excl)	P(incl|data)	P(excl|data)	BF_incl_
T	0.737	0.263	0.953	0.047	7.236
C	0.737	0.263	0.485	0.515	0.336
R	0.737	0.263	0.074	0.926	0.029
T*C	0.316	0.684	0.140	0.860	0.352
T*R	0.316	0.684	0.005	0.995	0.010
C*R	0.316	0.684	3.745e -4	1.000	8.117e -4
T*C*R	0.053	0.947	2.288e -6	1.000	4.118e -5

Note. T: MRI field strength (Tesla); C: MRI Contrast; R: Repetition; P(incl): prior inclusion probability; P(excl): prior exclusion probability; P(Incl|data): posterior inclusion probability; P(excl|data): posterior exclusion probability; BF_incl_: the inclusion Bayes factor.

Note that the results presented in section 3.2 and 3.2.1 are conceptually similar when a non-linear within-subject registration method is used (see Supplementary Fig. 1 and Supplementary Table 1 and 2).

### 3.3 Visibility of the STN

To test whether the observed differences in MNI space might be explained by differences in STN visibility, the STN was parcellated by two raters and the Dice coefficient was calculated to quantify the interrater reliability. The mean Dice coefficient was 0.70 (SD = 0.05) for 3 T and 0.61 (SD = 0.09) for the 7 T based masks. The Dice coefficient indicated moderate to substantial agreement between the two raters and were similar to our previous work (Keuken et al., 2017, Landis and Koch, 1977). The data was 4.95 (1/0.202) times more likely under the model with MRI field strength as a predictor compared to the Null model. In addition, we tested whether there were any differences in volume as quantified by the conjunction masks. The mean conjunction volume was 20.70 mm^3^ (SD = 7.16) for the 3 T and 15.09 mm^3^ (SD = 2.47) for the 7 T based masks. Note that the conjunction masks are considered extremely conservative volumetric estimates as only voxels that both raters agree on are included. The volumetric data was 2.93 (1/0.341) times more likely under the model with MRI field strength as a predictor than under the Null model.

## 4 Discussion

Direct targeting of the STN for DBS is shown to result in improved clinical outcome and has resulted in surgical centres to prefer it over an indirect targeting approach (Lahtinen et al., 2020, Machado et al., 2006, Tonge et al., 2016). However, the feasibility and accuracy of direct targeting is dependent on the quality of the MRI image (Hartmann et al., 2019, Machado et al., 2006). The current study assessed whether theoretical benefits of 7 T MRI translate into more reliable targeting of the STN for DBS. We did so by comparing neurosurgical targets across field strength, image modality and across repetition using a test–retest approach. Target accuracy was assessed by calculating the distance in millimetres between the repetitive target locations. We hypothesized that optimized 7 T image modalities would result in less variable target locations. Further, and in line with previous literature, we hypothesized that 7 T-QSM images would result in the least variability in targeting compared to any other 7 T images due to its superior ability in visualizing the STN.

The results, however, indicate that within these five patients there was substantial evidence that the test–retest reliability of neurosurgeons is not influenced by MRI field strength, contrast or targeting session. This indicates that the neurosurgeons selected the same target site within a given MRI contrast across sessions. It can therefore be argued that variability based on direct targeting methods probably is not a factor on itself in suboptimal placement of the DBS lead, since the same target site would have been selected if targeting was performed repetitively. Furthermore, it is interesting to note the anatomical variability between patients as shown in Fig. 4. This illustrates the importance of an individualized targeting approach which accounts for substantial anatomical variability as opposed to using indirect methods such as STN templates or standard coordinates (Alkemade et al., 2017, Cho et al., 2010, Duchin et al., 2018, Keuken et al., 2013)

Next to the variability in stereotactic planning, the exact anatomical location of the electrode target may potentially on itself be a factor in suboptimal placement of the DBS lead. The general consensus is that the effectiveness of DBS depends on the portion of the STN in which the DBS lead is placed, with the dorsolateral portion of the STN being most effective in treating PD (Duchin et al., 2018, Hamel et al., 2017, Starr, 2002, Welter et al., 2014). A second question that was addressed is whether the neurosurgeons select the similar target sites between MRI contrasts and field strengths, considering that different contrasts and resolutions might convey different anatomical information (Bazin et al., 2020, McRobbie et al., 2006, Visser et al., 2016a). While the neurosurgeons were stable in selecting the electrode location, the location itself seemed to differ between field strengths whereby the selected electrode location appeared (mainly) more ventral when using a 3 T MRI image versus a 7 T MRI image. This shift in location is unlikely to have occurred due to a difference in STN visibility as both 3 T and 7 T resulted in moderate to substantial interrater agreement. Note, however, that in the current study the visibility of the STN was not quantified by the neurosurgeons themselves, but by two independent anatomical experts and a third independent rater. As such, although unlikely, it cannot fully be ruled out that the neurosurgeons were hampered by reduced visibility of the STN. As such it does not seem that neurosurgeons are hampered by reduced visibility of the STN but it might be the case that they use different image features, such as landmarks, to determine the electrode location. Our results are conceptually in line with the recent work by (Bot et al., 2019) where it was shown that the intended DBS electrode sites were more posterior and inferior to the midcommissural point when using 1.5 T and 3 T compared to 7 T MRI images. Note, however, that another study failed to find differences in target location between the 3 T and 7 T MRI images (van Laar et al., 2016).

Whether the electrode is placed more ventral has clear clinical relevance as previous work has indicated that more ventral stimulation seems to be associated with reduced cognitive outcome (Machado et al., 2006, McNeely et al., 2011, Zarzycki and Domitrz, 2020). For example, it was shown that stimulation of specifically the ventral STN led to an impaired performance on the Go-No-Go task, which requires higher cognitive functions (Hershey et al., 2010). Our results showed that the selected electrode location using a 3 T MRI image is more ventral compared to using a 7 T MRI image. Future work should focus on whether this theoretical difference in STN targeting based on MRI strength actually leads to less cognitive and psychiatric side-effects. It should further be studied what differences in imaging features causes the difference in electrode location when targeting on 7 T versus 3 T MRI images.

There are a number of limitations to the present study. The number of patients that were included in the study was limited, but we feel that this is a minor issue as the main metric of interest was the test–retest reliability within a patient and that direct pre-operative planning approaches always employ individualized targeting (Isaacs et al., 2020b). Although the number of patients was limited, the main results were all supported by substantial or more evidence, inspiring reasonable confidence in our conclusions (Peter Rosenfeld and Olson, 2021, Schönbrodt and Wagenmakers, 2018). Another limitation is that the selection of MRI contrasts included a standard clinical 3 T protocol and an optimized 7 T protocol, adapted for anatomical changes with both age and disease. We did not however include either a 7 T-T2 or an optimized/quantitative 3 T-T2* based sequence which would have allowed for a direct comparison between field strengths while directly accounting for difference in MRI contrasts mechanism. As such, it remains challenging to disentangle the contributions of MRI contrast and MRI field strength in the difference in MNI target location. We attempted to quantify the different factors by conducting an analysis of effects where the results indicated that the data is 6.62 times more likely to occur in models that include MRI field strength than not, and that the data is 2.93 times more likely to occur under models that did not include MRI contrast as a predictor. Together with the findings reported by (Bot et al., 2019) we would tentatively interpret our results as evidence in favour of an effect of MRI field strength on the intended electrode position and not so much due to a difference in MRI contrast mechanisms. A final limitation that complicates the interpretability of the results in standard MNI space are the potential biases in MNI registrations for the 3 T data compared to the 7 T scans due to the difference in voxel geometry and volume (Mulder et al., 2019, Zhao et al., 2016).

In light of these limitations, the present study provides substantial evidence that regardless of the MRI field strength and MRI contrast, neurosurgeons are stable in selecting the intended DBS electrode location. In addition, we conclude that the intended electrode location differs between MRI field strengths, where the 3 T scans resulted in a more ventral location. Future research should focus on what image features drive the neurosurgeons to select a slightly different location across the images.

## CRediT authorship contribution statement

**BRI, MH:** Conceptualization, Data curation, Formal analysis, Methodology, Project administration, Software, Visualization, Writing - original draft, Writing - review & editing. **MLK:** Data curation, Supervision, Writing - review & editing. **PLK, LA:** Data curation, Writing - review & editing. **YT:** Data curation, Funding acquisition, Writing - review & editing. **MCK:** Formal analysis, Methodology, Software, Visualization, Writing - original draft, Writing- review & editing. **BUF:** Funding acquisition, Supervision, Writing - review & editing.

## Declaration of Competing Interest

The authors declare that they have no known competing financial interests or personal relationships that could have appeared to influence the work reported in this paper.
