# Supplementary material for: Variability in subthalamic nucleus targeting for deep brain stimulation with 3 and 7 Tesla magnetic resonance imaging
Source: Neuroimage Clin. 2021 Sep 16;32:102829. doi: 10.1016/j.nicl.2021.102829 (PMC8463907; doi:10.1016/j.nicl.2021.102829)
Supplement: Supplementary data 1 [file mmc1.docx]

Supplementary Figure 1:

*
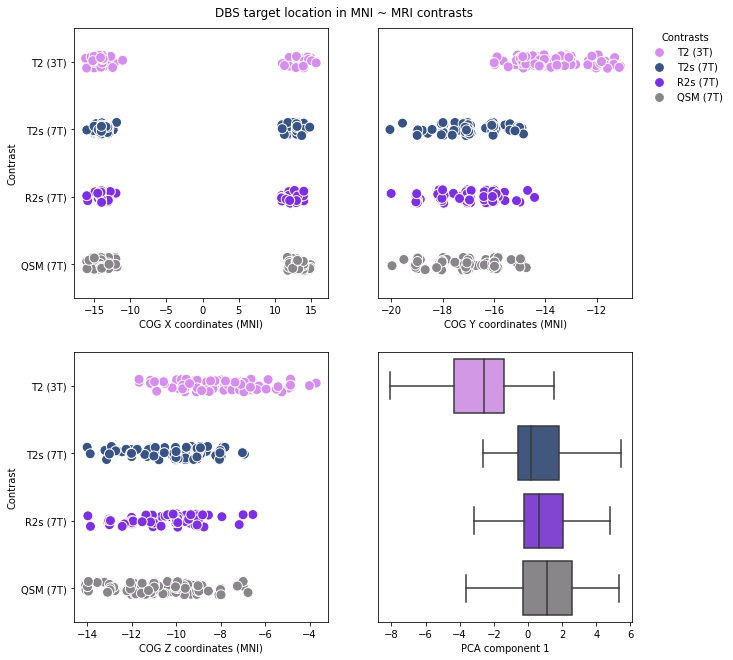
*

*Supplementary Figure 1. The intended DBS electrode location in MNI space over MRI contrast when using a fully non-linear registration pipeline. Similar to Figure 4 in the main document, the three scatterplot panels show the X, Y, and Z MNI coordinate of the planned electrode’s Centre of Gravity (COG) per MRI contrast, patient, surgeon and planning session registered from native to MNI space. The difference with Figure 4 is that the individual slab to individual whole brain registration was done using a non-linear symmetric normalization function as opposed to a purely rigid transformation. As a result, not only does the COG clearly differ on the Y axis but now also on the Z axis for the 3T T2 contrast relative to the 7T contrasts.*

Supplementary Table 1:

| *Supplementary table 1. Model comparison of the spatial location of the DBS electrode target, non-linear registration pipeline.* | | | | | | | | | | | |
| --- | --- | --- | --- | --- | --- | --- | --- | --- | --- | --- | --- |
| **Models** | | **P(M)** | | **P(M\|data)** | | **BF _M_** | | **BF _10_** | | **error %** | |
| T |  | 0.053 |  | 0.577 |  | 24.524 |  | 1.000 |  |  |  |
| T + C |  | 0.053 |  | 0.230 |  | 5.377 |  | 0.399 |  | 8.262 |  |
| T + C + T  ✻  C |  | 0.053 |  | 0.105 |  | 2.103 |  | 0.181 |  | 5.662 |  |
| T + R |  | 0.053 |  | 0.036 |  | 0.671 |  | 0.062 |  | 4.000 |  |
| C |  | 0.053 |  | 0.026 |  | 0.487 |  | 0.046 |  | 3.859 |  |
| T + C + R |  | 0.053 |  | 0.013 |  | 0.241 |  | 0.023 |  | 4.015 |  |
| T + C + R + T  ✻  C |  | 0.053 |  | 0.007 |  | 0.128 |  | 0.012 |  | 7.690 |  |
| T + R + T  ✻  R |  | 0.053 |  | 0.003 |  | 0.049 |  | 0.005 |  | 5.196 |  |
| C + R |  | 0.053 |  | 0.002 |  | 0.029 |  | 0.003 |  | 3.429 |  |
| T + C + R + T  ✻  R |  | 0.053 |  | 9.530e  -4 |  | 0.017 |  | 0.002 |  | 4.180 |  |
| T + C + R + T  ✻  C + T  ✻  R |  | 0.053 |  | 4.264e  -4 |  | 0.008 |  | 7.394e  -4 |  | 4.437 |  |
| T + C + R + C  ✻  R |  | 0.053 |  | 1.979e  -4 |  | 0.004 |  | 3.431e  -4 |  | 4.342 |  |
| T + C + R + T  ✻  C + C  ✻  R |  | 0.053 |  | 9.010e  -5 |  | 0.002 |  | 1.562e  -4 |  | 4.209 |  |
| T + C + R + T  ✻  R + C  ✻  R |  | 0.053 |  | 3.510e  -5 |  | 6.318e  -4 |  | 6.086e  -5 |  | 7.064 |  |
| C + R + C  ✻  R |  | 0.053 |  | 2.430e  -5 |  | 4.374e  -4 |  | 4.214e  -5 |  | 4.400 |  |
| T + C + R + T  ✻  C + T  ✻  R + C  ✻  R |  | 0.053 |  | 1.509e  -5 |  | 2.716e  -4 |  | 2.617e  -5 |  | 5.723 |  |
| T + C + R + T  ✻  C + T  ✻  R + C  ✻  R + T  ✻  C  ✻  R |  | 0.053 |  | 2.918e  -6 |  | 5.253e  -5 |  | 5.060e  -6 |  | 11.018 |  |
| Null model (incl. PatientNr, Surgeon) |  | 0.053 |  | 1.940e -52 |  | 3.492e -51 |  | 3.363e -52 |  | 2.722 |  |
| R |  | 0.053 |  | 7.849e -54 |  | 1.413e -52 |  | 1.361e -53 |  | 3.696 |  |
|  | | | | | | | | | | | |
| *Note. All models include PatientNr, Surgeon. T: MRI field strength (Tesla); C: MRI Contrast; R: Repetition; P(M): Prior model probability; P(M\|data): posterior model probability; BFM: the change from prior odds to posterior odds; BF10: the Bayes factor relative to the best model; error %: indicates the precision of the numerical approximation and it is thought that in many situations an error percentage below 20.0% is acceptable (Bergh et al., 2020).* | | | | | | | | | | | |

Supplementary Table 2:

| *Supplementary table 2. Analysis of Effects – Spatial location of the DBS electrode targets, non-linear registration pipeline.* | | | | | | | | | | | |
| --- | --- | --- | --- | --- | --- | --- | --- | --- | --- | --- | --- |
| **Effects** | | **P(incl)** | | **P(excl)** | | **P(incl\|data)** | | **P(excl\|data)** | | **BF _incl_** | |
| T |  | 0.737 |  | 0.263 |  | 0.972 |  | 0.028 |  | 12.414 |  |
| C |  | 0.737 |  | 0.263 |  | 0.385 |  | 0.615 |  | 0.223 |  |
| R |  | 0.737 |  | 0.263 |  | 0.062 |  | 0.938 |  | 0.024 |  |
| T✻C |  | 0.316 |  | 0.684 |  | 0.112 |  | 0.888 |  | 0.274 |  |
| T✻R |  | 0.316 |  | 0.684 |  | 0.004 |  | 0.996 |  | 0.009 |  |
| C✻R |  | 0.316 |  | 0.684 |  | 3.654e -4 |  | 1.000 |  | 7.920e -4 |  |
| T✻C✻R |  | 0.053 |  | 0.947 |  | 2.918e -6 |  | 1.000 |  | 5.253e -5 |  |
|  | | | | | | | | | | | |

*Note. T: MRI field strength (Tesla); C: MRI C; R: Repetition; P(incl): prior inclusion probability; P(excl): prior exclusion probability; P(Incl|data): posterior inclusion probability; P(excl|data): posterior exclusion probability; BFincl: the inclusion Bayes factor.*
